# Treadmill exercise pretreatment ameliorated structural synaptic plasticity impairments of medial prefrontal cortex in vascular dementia rat and improved recognition memory

**DOI:** 10.1038/s41598-024-57080-4

**Published:** 2024-03-26

**Authors:** Linlin Zhang, Yuanyuan Chen, Yongzhao Fan, Lin Shi

**Affiliations:** 1 Department of Physical Education, Henan Normal University, Xinxiang, 453007 China; 2 Department of Psychology and Education, Shantou Polytechnic, Shantou, 515071 China; 3 Department of Physical Education and Sport, Shanghai Ocean University, Shanghai, 201306 China

**Keywords:** Treadmill exercise, Vascular dementia, Synaptic plasticity, Recognition memory, Physiology, Psychology, Diseases, Neurology

## Abstract

This study aimed to investigate structural synaptic plasticity in the medial prefrontal cortex of rats under treadmill exercise pretreatment or naive conditions in a vascular dementia model, followed by recognition memory performance in a novel object recognition task. In this study, 24 Sprague–Dawley rats were obtained and randomly assigned into 4 groups as follows: control group (Con group, n = 6), vascular dementia (VD group, n = 6), exercise and vascular dementia group (Exe + VD group, n = 6), and exercise group (Exe group, n = 6). Initially, 4 weeks of treadmill exercise intervention was administered to the rats in the Exe + VD and Exe groups. Then, to establish the vascular dementia model, the rats both in the VD and Exe + VD groups were subjected to bilateral common carotids arteries surgery. One week later, open-field task and novel recognition memory task were adopted to evaluate anxiety-like behavior and recognition memory in each group. Then, immunofluorescence and Golgi staining were used to evaluate neuronal number and spine density in the rat medial prefrontal cortex. Transmission electron microscopy was used to observe the synaptic ultrastructure. Finally, microdialysis coupled with high-performance liquid chromatography was used to assess the levels of 5-HT and dopamine in the medial prefrontal cortex. The behavior results showed that 4 weeks of treadmill exercise pretreatment significantly alleviated recognition memory impairment and anxiety-like behavior in VD rats (*P* < 0.01), while the rats in VD group exhibited impaired recognition memory and anxiety-like behavior when compared with the Con group (*P* < 0.001). Additionally, NeuN immunostaining results revealed a significant decrease of NeuN-marked neuron in the VD group compared to Con group (*P* < 0.01), but a significantly increase in this molecular marker was found in the Exe + VD group compared to the Con group (*P* < 0.01). Golgi staining results showed that the medial prefrontal cortex neurons in the VD group displayed fewer dendritic spines than those in the Con group (*P* < 0.01), and there were more spines on the dendrites of medial prefrontal cortex cells in Exe + VD rats than in VD rats (*P* < 0.01). Transmission electron microscopy further revealed that there was a significant reduction of synapses intensity in the medial prefrontal cortex of rats in the VD group when compared with the Con group(*P* < 0.01), but physical exercise was found to significantly increased synapses intensity in the VD model (*P* < 0.01). Lastly, the levels of dopamine and 5-HT in the medial prefrontal cortex of rats in the VD group was significantly lower compared to the Con group (*P* < 0.01), and treadmill exercise was shown to significantly increased the levels of dopamine and 5-HT in the VD rats (*P* < 0.05). Treadmill exercise pretreatment ameliorated structural synaptic plasticity impairments of medial prefrontal cortex in VD rat and improved recognition memory.

## Introduction

VD involve the remarkable vascular cognitive impairment cause by multiple vascular factors. In the early and developmental stages of VD, it most often characterized by various degrees cognitive decline^1^. Neurobiologists have made several attempts to elucidate the mechanism underlying the VD-induced memory impairment^2,3^. Accumulating evidence aimed to elucidate the link between VD and memory function has focused on synaptic plasticity, which could also be a potential target for treatment interventions. Synaptic plasticity, or the change in connections between neurons caused by experience, underpins learning and memory in the brain^4^. VD can induce a deficit of synaptic plasticity in the hippocampus^5,6^, striatum^7^ and corpus callosum^8^ regions, such as the decrease of dendritic spine density and synaptic proteins. It has been known for a few years that acupuncture^9^, social interaction^10^ and exercise^11^ have a positive influence in patients with VD. It is worth mentioning that exercise can not only ameliorate VD symptoms, but also prevent the development at an early stage of VD^12,13^. Furthermore, there is evidence supporting the conclusion that the neuroprotective effect of exercise in a VD model^14^. However, it remains to be clarified whether exercise has a positive effect in medial prefrontal cortex regions.

Recent research advances in humans^15^ and animals^16^ have demonstrated that physical exercise has a significant effect on learning and memory. There is growing evidence that exercise can enhance hippocampal neurogenesis in normal^17^ or diseases states, such as APP/PS1 transgenic^18^, stress^19^, and VD models^20^. To date, an increasing number of researchers have further explored the mechanism underlying exercise-induced memory improvement. Structural synaptic plasticity, which is necessary for memory formation during brain development, provides a possible explanation^21^. Exercise is a non-drug therapy that has proved to be beneficial for increasing structural synaptic plasticity in different regions of the brain^22–24^. As an important region in the brain, the medial prefrontal cortex is closely related to learning memory and execution function^25^. It has a high neuronal density and metabolic rate, marking it susceptible to ischemia and hypoxia injury^26,27^. Prefrontal cortex injury caused by prolonged bilateral common carotid artery ligation can significantly affect higher brain functions, such as learning and memory functions^26,28^. However, no previous research has investigated the effect of exercise on VD via structural synaptic plasticity in the prefrontal cortex region. Therefore, the current study sought to determine whether treadmill exercise pretreatment can alleviate recognition memory impairment by regulating structural synaptic plasticity parameters of the prefrontal cortex in a VD rat model.

## Materials and methods

### Experimental animals and grouping

Sprague–Dawley rats (males 200–225 g; Shanghai SLAC Laboratory Animal Co., Ltd) were housed (three per cage; cage size:460 × 300 × 160 mm) socially in a controlled room (Temperature: 22 ± 3 °C; Humidity: 40 to 70%) on a reversed 12 h light–dark cycle (light off at 6 AM). Water and food were made available all the time. All the animal procedures and methods conducted in this study were approved by the Ethics Committee of Experimental Animals of Capital University of Physical Education and Sport (Approval number 2020A76). We confirm that all methods were performed in accordance with the relevant guidelines and regulations and ARRIVE guidelines。

**Figure 1:**
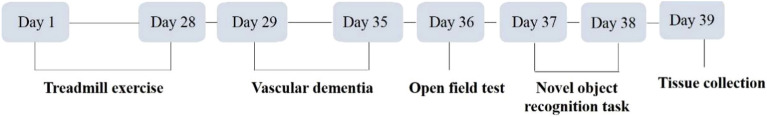
Schematic timeline of study design.

Rats in this experiment were given a one-week period to adapt to their environment. Then, the rats (n = 24) were randomly divided into 4 groups as follows: control group (Con group, n = 6), VD group (n = 6), exercise and vascular dementia group (Exe- VD group, n = 6), and exercise group (Exe group, n = 6). The treadmill exercise interventions and the establishment of VD model are in accordance with Fig. 1. Regarding the behavioral task, all the animals were subjected to an open-field task and a novel recognition memory task. Subsequently, all the animals were sacrificed for further assays. NeuN immunostaining was used to evaluate the quantity of positive cells in the medial prefrontal cortex. Golgi staining was used to analyze morphological alterations in neuronal dendritic spines. Transmission electron microscopy was used for in vivo synaptic imaging. Microdialysis coupled with high-performance liquid chromatography was used to analyze the level of 5-HT and dopamine in the medial prefrontal cortex.

### Treadmill exercise pretreatment

In this study, treadmill exercise was applied for 4 weeks. Thus, the rats in the Exe + VD and Exe groups were forced to run on a treadmill device (BHW-PT/5 s, Anhui, China) for 4 weeks. All the rats involved in the intervention groups were conditioned for treadmill exercise for 30 min on 3 consecutive days (at 8 m/min on the first day and 12 m/min on the second and third days). Subsequently, the rats were subjected to a treadmill exercise pretreatment on a 0° slope, starting with a speed of 12 m/min for 10 min. Then, this was scaled up to 15 m/min for 50 min. In sum, all the rats in the Exe + VD and Exe groups were trained for an hour per day (6 PM to 10 PM), 5 days per week, for a total of 4 weeks. According to previous study, the intensity of treadmill exercise in this experimentwas categorized as low intensity exercise^29^. The running speed suitable for treadmill exercise was determined based on our previous studies that showed protective effects against AD and other brain diseases^29,30^.

### VD model

The VD model was established using the two vessel occlusion method according to previous studies^31,32^. First, the animals in the VD and Exe + VD groups were anesthetized with an intraperitoneal (IP) injection of 1% pentobarbital sodium (60 mg/kg). Regarding the two-vessel occlusion procedure, the skin of the neck was prepped with alcohol. Then, a ventral midline cervical incision was made, through which we identified and isolated the bilateral common carotid arteries (BCCAs) from the vagus. Subsequently, BCCAs were gently isolated and ligated with 10–0 silk sutures. The wound area was treated locally with penicillina after the incision was sutured. Afterwards, the general condition and body weight of each rat were monitored for the next 3 days. The mortality rate after BCCA occlusion in this study is 14.29%. After 1 week, the “Zea-Longa” 5-point scale was used to confirm whether the animal model had been established successfully. The success rate of establishing VD rat model is 92.31%.

### Open-field test

The open-field test (OFT) measures spontaneous locomotor activity as well as anxiety-like behaviors^33^. This test was conducted with apparatus being placed on a square arena (Black; 100 cm × 100 cm × 50 cm), and the bottom of the arena was divided into two zones: an outside and a center zone. At the beginning of testing, the animal was placed in a particular corner of the area, and its behavior was recorded over 10 min. Then, 70% ethanol was used to clean the test apparatus, followed by drying with paper towels, and the rat were returned to their home cage. In this test, total distance, time traveled in every zone, number of entries into every zone and were recorded.

### Novel object recognition task

The recognition memory and the behavior to explore a new environment were evaluated via the novel object recognition task^34^. The rats were allowed to freely explore two identical objects for 10 min on the first day. The test phase began after a delay of 5 min (short-term recognition memory) or 24 h (long-term recognition memory), during which one of the objects was replaced by a novel one (similar size texture and color, but different in shape), and the rats were allowed to explore it for 10 min. Under normal states, rats often tend to spend more time examining unfamiliar objects. As a result, the time spent by a rat in exploring the novel object was used as a measure of memory. Thus, the novel object discrimination index was determined by dividing the time interacting with the novel object by the total time explored both objects (i.e., [Time Novel/(Time Novel + Time Familiar)] × 100%).

### Immunofluorescence

Regarding NeuN immunostaining, the number of positive cells in the medial medial prefrontal cortex region was counted. After perfusion with saline and 4% paraformaldehyde, the whole brain tissues were quickly removed, and the medial prefrontal cortex block were fixed in 4% paraformaldehyde overnight at 4 °C and cryoprotected in sucrose. Coronal sections were cut at 30 μm thickness using a cryostat. Then, the coronal sections were subjected to fluorescence immunostaining using primary antibodies (rabbit monoclonal to NeuN—Neuronal marker, 1:200, ab177487, Abcam) at 4 °C overnight, followed by secondary antibody staining (Alexa Fluor 488-conjugated goat anti-Rabbit IgG, 1:1000, ab150077, Abcam) to verify neuron expression. Images were acquired with a confocal fluorescence microscope (Olympus) under 400 × magnification. All the measurements for immunofluorescent observation were performed in 3 rats from each group.

### Golgi staining

The FD Rapid Golgi Stain™ Kit (FD Neurotechnologies, Inc) was used to perform Golgi staining. This can evaluate minimal morphological changes in neuronal dendrites and dendritic spines^35^. Briefly, isolated tissue blocks (prefrontal cortex) were rinsed quickly with PBS and immersed in the fixative for 48 h. Then, tissue blocks were added to the Golgi-cox staining solution and incubated for 14 days (new staining solution was changed after 48 h, and then every 3 days). After treatment with 80% glacial acetic acid and 30% sucrose, the tissue blocks were sliced into sections of 100 microns using an oscillating microtome and pasted on a gelatin slide. Then, the tissue blocks were hardened and fixed with acid for 15 min, distilled water was used to wash for 3 min,

and the sections were dried and sealed using glycerin gelatin. Finally, a digital slice scanner (Nikon DS-U3) was used to acquire panoramic images of brain tissue, which was analyzed using ImageJ (Fiji) software.

### Transmission electron microscopy

For the quantification of medial prefrontal cortex synaptic changes, transmission electron microscopy was used to analyze the number of synapses^36^. To analyze synaptosomes, the medial prefrontal cortex block (no more than 1 mm^3^) was washed with PBS and immediately placed into an EP tube containing TEM fixative at 4 °C. Then, the tissue was fixed using 1% OsO4 in 0.1 M PB (pH 7.4) at room temperature for 7 h, and rinsed 3 times in 0.1 M PB (pH 7.4). Dehydration was performed at room temperature using 30–100% ethanol and pure acetone for 1 h. Subsequently, the tissue was subjected to resin penetration and embedding as well as stored overnight in an oven at 37 °C. The tissue embedding model was transferred to a hotter oven (65 °C) and allowed to polymerize for  > 48 h. Then, the tissue was sectioned into 60–80 nm slices using an ultra-microtome (Leica UC7) and collected on copper grids, followed by examination with transmission electron microscopy (Hitachi). The number of synapse was counted under the 200 × magnification and calculated by ImageJ.

### High-performance liquid chromatography for DA tissue content

After behavior test, animals were placed in a stereotaxic apparatus to ensure the skull is at the same level. Then, the bregma is regarded as origin of coordinates, microdialysis (MD) probe was stereotaxically implanted in the rat medial prefrontal cortex (A/P, + 3.2 mm; M/L, ± 0.6 mm and D/V, − 1.5 mm from dura), and extracellular fluid was collected in the EP tubes. When obtained extracellular fluid of each rats, high-performance liquid chromatography with electrochemical detection was used to determine the levels of 5-HT and DA in extracellular fluid. Before the analysis, the samples were diluted with ice-cold mobile phase that was deoxygenated with argon in a 1:10 ratio, followed by direct injection into the high-performance liquid chromatography device^37^. The mobile phase was flowing at a rate of 0.5 mL/min.

### Data analysis

Data in this experiment were summarized as mean ± SEM. All statistical analyses were performed by GraphPad Prism software (GraphPad Software, La Jolla, CA, USA). One-way analysis of variance was used to compare means among multiple groups, followed by Tukey’s post hoc test. *P* < 0.05 was chosen as the level of statistical significance.

## Results

### Treadmill exercise pretreatment ameliorated anxiety behavior in VD rat

**Figure 2:**
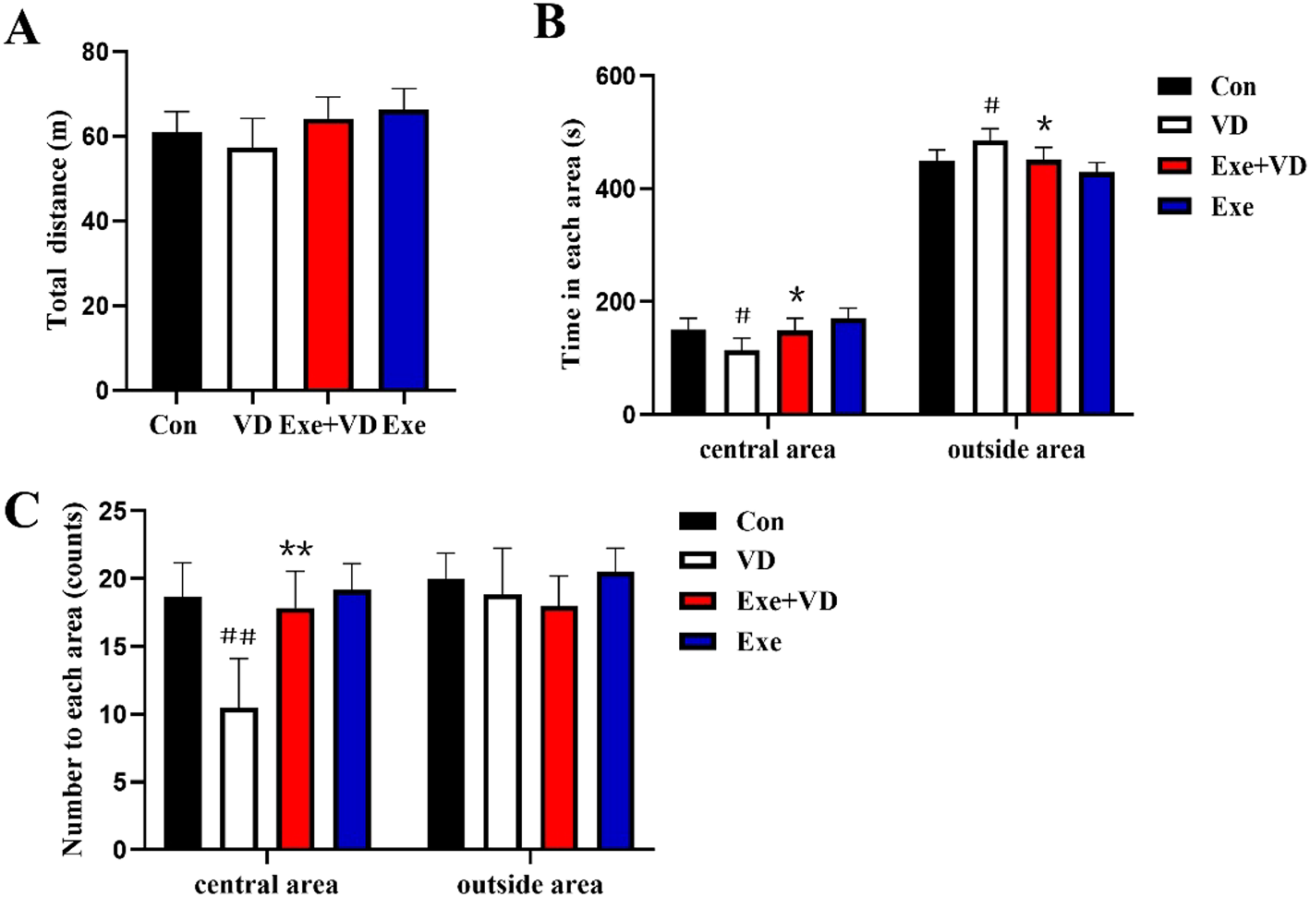
Treadmill exercise prevented anxiety- like behavior after VD in open field test. The figure shows total distance traveled (**A**)**,** time traveled in each area (**B**), number of entries into each area (**C**)**.** N = 6/group. ^#^*P* < 0.05 and ^##^*P* < 0.01 vs. Con group; *P < 0.05 and **P < 0.01 vs. VD group.

Results were obtained for all behavioral parameters in the OFT (Fig. 2). There was no significant difference in total distance traveled among the four groups (*P* > 0.001; Fig. 2A). In terms of the time spent in the central zone, the rats in the VD group spent significantly lesser time than those in the Con group (*P* < 0.001; Fig. 2B). Moreover, the rats in the Exe + VD group spent significantly more time in the central zone than those in the VD group (*P* < 0.001; Fig. 2B).

In addition, rats in the VD group had fewer number of times to enter the central area than those in the Con group (*P* < 0.01; Fig. 2C). After treadmill exercise pretreatment, there was a significantly increased number of times to enter the central area in the Exe + VD rats when compared with VD group (*P* < 0.01, *P* < 0.001; Fig. 2C).

### Treadmill exercise pretreatment ameliorated recognition memory impairment in VD rat

**Figure 3:**
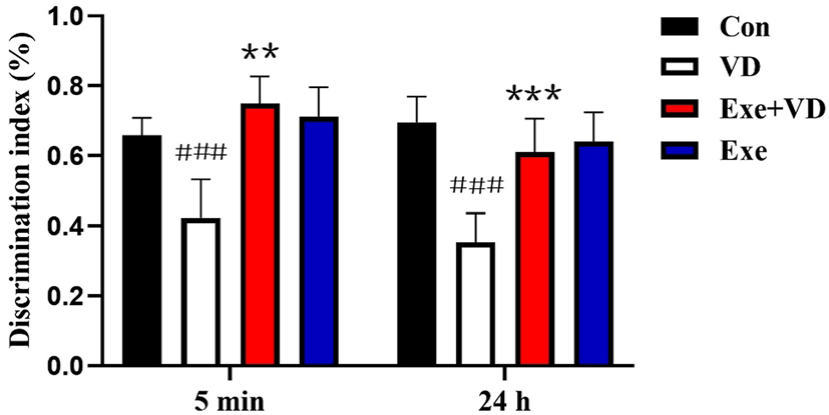
Discrimination index in test phase of novel object recognition task. N = 6/group. ^###^*P* < 0.001vs. Con group; **P < 0.01 and ***P < 0.001 vs. VD group.

To assess novelty discrimination, a novel object recognition task was adopted in each group. In this test, the discrimination index of rats was significantly lower in the VD group as compared to the Con group (*P* < 0.001; Fig. 3), which indicating impaired object discrimination of VD rats. Conversely, the discrimination index of rats in the Exe + VD group was significantly greater than that of rats in the VD group (*P* < 0.01; Fig. 3), which suggests the VD rats have a positive trend of object recognition after exercise interventions. Thus, these results suggest that treadmill exercise pretreatment can improve VD-induced recognition memory for objects that had been previously encountered.

### Treadmill exercise pretreatment enhanced neuron density of medial prefrontal cortex in VD rat

**Figure 4:**
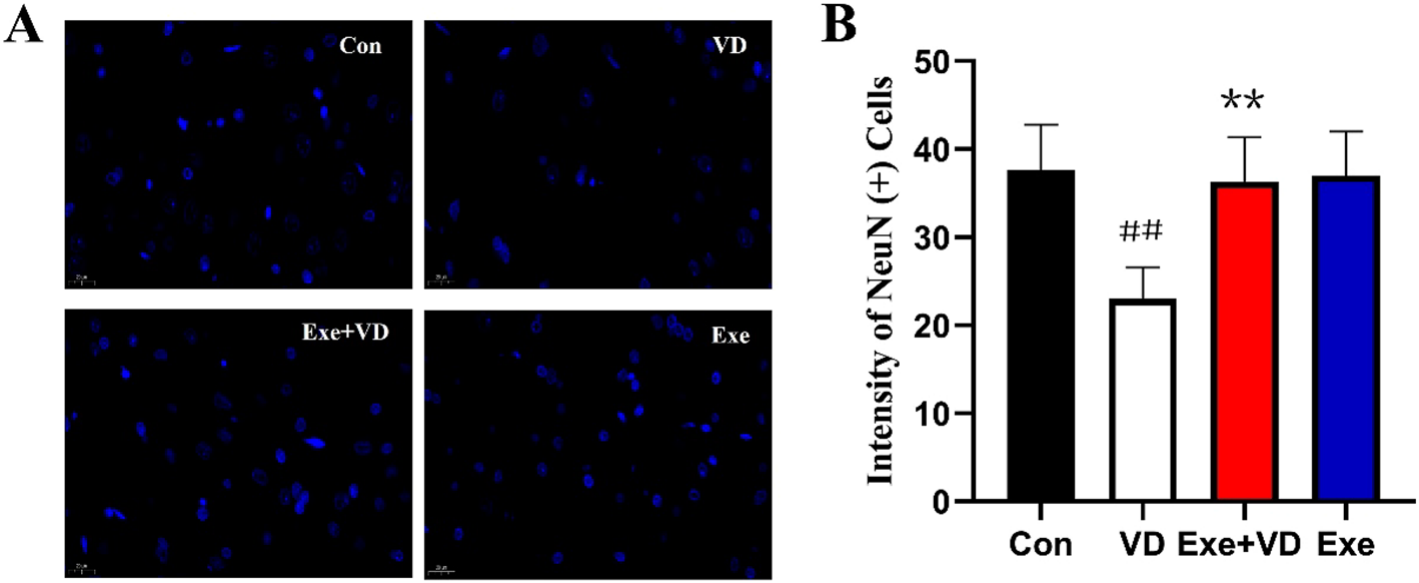
Quantification analysis of single immunofluorescent staining with mouse anti-NeuN primary.. N = 3/group. ^##^*P* < 0.01 vs. Con group; **P < 0.01 vs. VD group.

To investigate whether there is an association between recognition memory restoration via treadmill exercise pretreatment restored in recognition memory and structural synaptic plasticity, we first counted the number of neurons in the cortex necessary for the transmission of information linked to memory and learning. NeuN immunostaining was conducted to examine the expression of NeuN-marked neurons in rat medial prefrontal cortex from the four groups listed in Fig. 4A. The results showed a significant decrease in NeuN-marked intensity in Con group when compared to VD group (*P* < 0.01; Fig. 4B). Regarding the effects of treadmill exercise on NeuN expression in the VD model, we found a significant increase in this molecular marker in rats that received a systemic treadmill exercise compared to the Con group (*P* < 0.01; Fig. 4B). Thus, we concluded that treadmill exercise pretreatment can ameliorate VD-induced the decrese of neuron intensity in the medial prefrontal cortex.

### Treadmill exercise pretreatment enhanced the spine density of the medial prefrontal cortex in VD rat

**Figure 5:**
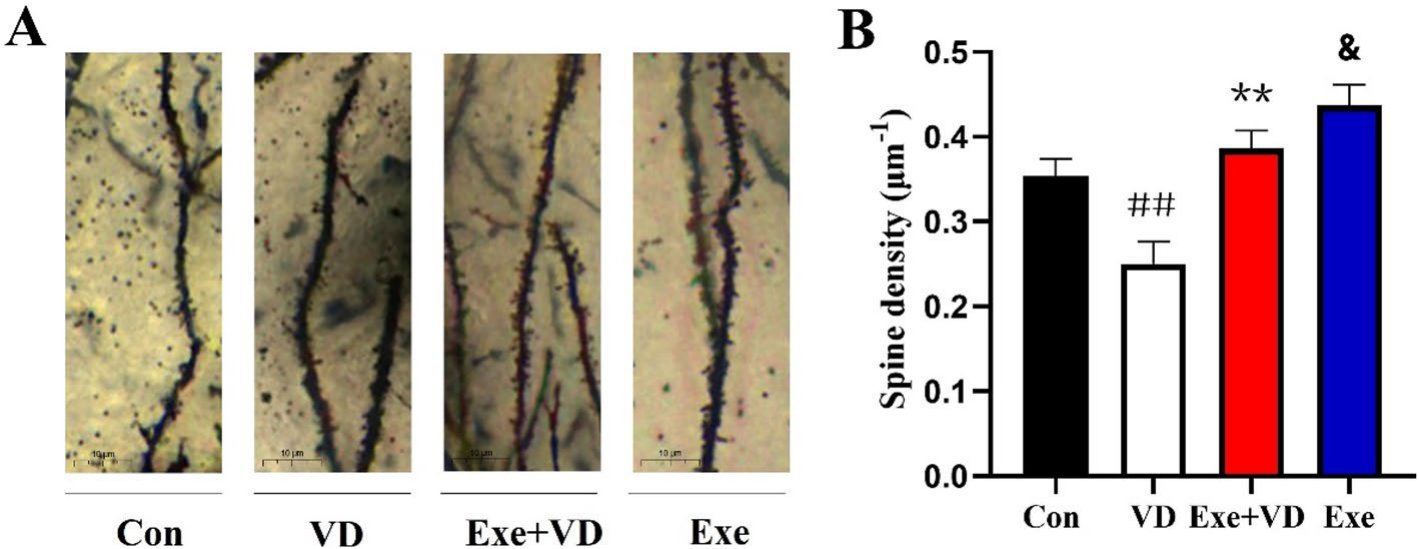
(**A**) Golgi-stained basal dendrites of medial prefrontal cortex pyramidal neurons in each group. (**B**) Quantification of mature spine density of the dendritic segments. N = 3/group. ^##^*P* < 0.01 vs. Con group; **P < 0.01 vs. VD group; ^&^*P* < 0.01 vs. Con group.

Golgi staining was performed to investigate whether treadmill exercise pretreatment alters the manifestation of dendritic spine in VD brain tissue, which are presented in Fig. 5A. We found that the medial prefrontal cortex neurons in the VD group revealed a significantly lower quantity of dendritic spines than those in the Con group (*P* < 0.01; Fig. 5B). Conversely, significantly differences were observed of the dendritic spine number in the Exe + VD group when compared with VD group. (*P* < 0.01; Fig. 5B). Thus, treadmill exercise pretreatment can inhibited VD-induced impairment of dendritic spines in the medial prefrontal cortex.

### Treadmill exercise pretreatment inhibited ultrastructure impairment of the medial prefrontal cortex in VD rat

**Figure 6:**
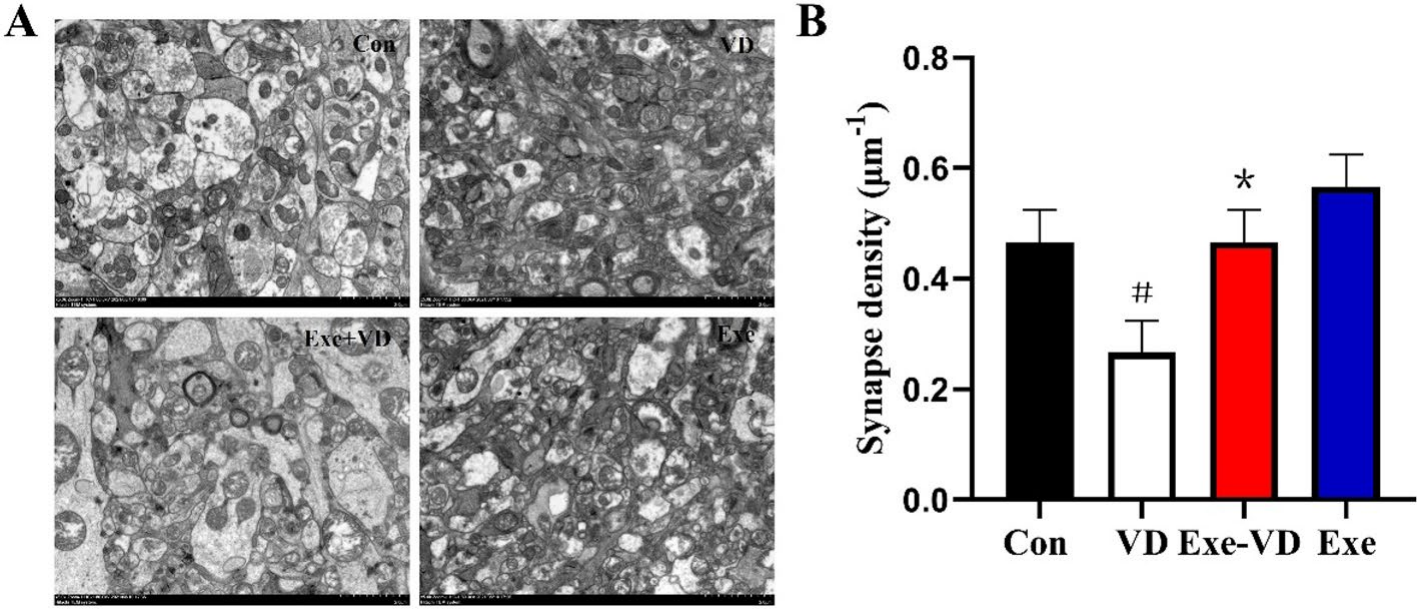
Representative images of ultrastructure from the medial prefrontal cortex neurons (**A**) and quantitative analysis of the density of synapse (**B**) are shown. The synapses are marked by the blue arrowheads. N = 3/group. ^#^*P* < 0.05 vs. Con group; *P < 0.05 vs. VD group.

We quantified the number of synapses in the medial prefrontal cortex, which are necessary for the establishment of recognition memory (Fig. 6A). The results of transmission electron microscopy revealed a significant reduction in the synapse density in the medial prefrontal cortex of rats in the VD group compared to the Con group (*P* < 0.05; Fig. 6B). Conversely, there was a significant increase in the synapse density in the medial prefrontal cortex of VD rats following the treadmill exercise pretreatment (*P* < 0.05; Fig. 6B). These data supported the hypothesis that treadmill exercise pretreatment can increase the synapse density in the medial prefrontal cortex of VD rats.

### Treadmill exercise pretreatment inhibited the decrease of medial prefrontal cortex dopamine and 5-HT in VD rat

**Figure 7:**
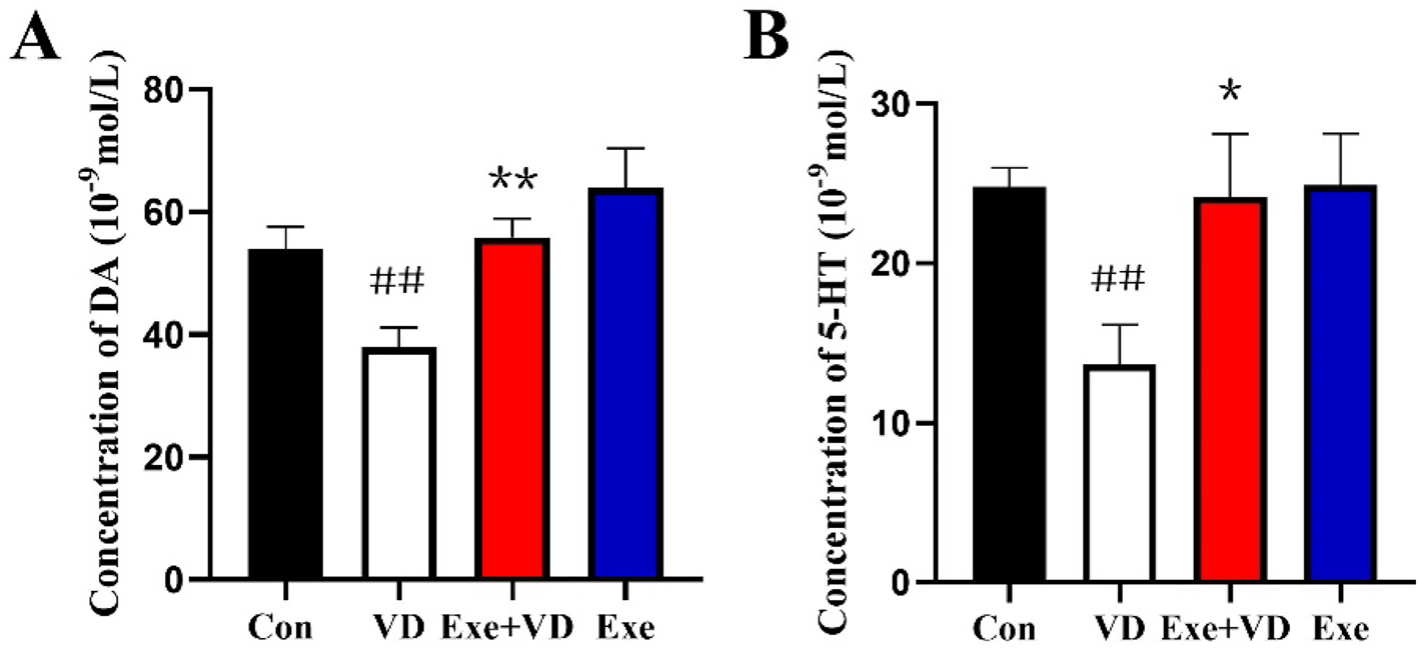
Dynamic changes of medial prefrontal cortex dopamine and 5-HT in four group. A: Dopamine B:5-HT. N = 3/group. ^##^*P* < 0.01 vs. Con group; *P < 0.05 and **P < 0.01 vs. VD group.

We observed dynamic changes in medial prefrontal cortex dopamine and 5-HT in the four groups. Our results revealed that the level of medial prefrontal cortex dopamine and 5-HT in the VD group was significantly lower when compared to the Con group (*P* < 0.01; Fig. 7). On the contrary, the level of medial prefrontal cortex dopamine and 5-HT in the Exe + VD group was significantly higher compared to the VD group, (*P* < 0.05; Fig. 7). The results indicated that treadmill exercise pretreatment can increase the level of medial prefrontal cortex dopamine and 5-HT in VD rats.

## Discussion

Our recent study demonstrated that VD rats exhibited impaired recognition memory, while treadmill exercise pretreatment was found to prevent recognition memory impairment by enhancing structural synaptic plasticity in a VD model. Moreover, we demonstrated that in a VD rat model established by prolonged two-vessel occlusion, treadmill exercise pretreatment ameliorates recognition memory impairment and anxiety-like behavior in a novel object recognition task and an open-field test. Following the treadmill exercise pretreatment, there was a resultant increase in neurons, dendritic spines density, density of synapses, and repair of structural synaptic plasticity of the medial prefrontal cortex in VD rats. Therefore, a possible mechanism through which treadmill exercise ameliorates recognition memory impairment and anxiety-like behavior could be by strengthening structural synaptic plasticity of the medial prefrontal cortex.

In the behavior test, after subjecting the rats in each group to behavioral tests (novel object recognition tasks and open-field), we found that the discrimination index of recognition memory was significantly decreased in the VD group compared to the Con group. Recognition memory is often an essential part of cognitive function. As mentioned above, the two-vessel occlusion-induced VD rat model is characterized by damage to the hippocampus and medial prefrontal cortex, caused by prolonged cerebral hypoperfusion, which results in cognitive deficits. As the VD disease progresses, the patients gradually experience recognition memory impairment leading to logical memory impairments and declarative metamemory disorders. Moreover, VD patients were found to exhibited anxiety-like behavior^38^, and this phenomenon was also found in our study and others animal studies^6,39^. Thus, we suggested that significant recognition memory impairment and anxiety-like behavior were present in the VD model. We also found that the discrimination index of rats in the Exe + VD group was significantly greater than that of rats in the VD group. This indicates that the rats in the Exe + VD group could recognize the presence of a novel object and exhibited recall the memory of an object previously observed. This result suggested that 4-week treadmill exercise pretreatment can ameliorate VD-induced recognition memory impairment and anxiety-like behavior in novel recognition memory and open-field tasks. Thus, improvement of recognition memory is a potential target for interventions against VD. There is currently a high interest in research about the role of exercise in reducing cognitive impairment and anxiety-like conditions. Previous studies demonstrated that exercise reverses recognition memory impairment and anxiety-like behavior in VD rats model^40,41^. Thus, our results showed that 4 weeks of treadmill exercise pretreatment ameliorates recognition memory impairment and anxiety-like behavior caused by two-vessel occlusion to some extent in VD rats. Although we have demonstrated that treadmill exercise pretreatment is involved in alleviating recognition memory impairment and anxiety-like behaviors, the potential mechanisms of the positive effects of treadmill exercise pretreatment on recognition memory in VD rat models needs to be further investigated.

Next, the potential underlying mechanism through which treadmill exercise induced an improvement of recognition memory in the VD rat model was examined. The morphological basis of memory is structural synaptic plasticity, which also plays important roles in learning and memory formation^42,43^. Learning and memory requires neural adaptations and dendritic spine participation, which are thought to be mediated by activity-dependent structural synaptic plasticity^44^. Therefore, the neuronal impairment will likely cause changes in structural synaptic plasticity. Previous studies demonstrated via Nissl staining that VD rats displayed significantly neuron structural changes in the hippocampal^45^. The immunostaining results in our study demonstrated that treadmill exercise pretreatment can ameliorate VD-induced the decrease of neuron density in the medial prefrontal cortex region of VD rats, as determined by NeuN immunostaining. In addition to NeuN (which is considered a nuclear marker), we also detected fluorescence levels higher than the threshold in the neurons from the rat brains^46^. Thus, we concluded that there was a significant decrease in medial prefrontal cortex neurons in the VD rats, which were restored by treadmill exercise pretreatment. the results by *golgi staining* also demonstrated that 4 weeks of treadmill exercise pretreatment can ameliorate VD-induced impairment of dendritic spines in the medial prefrontal cortex. As small protrusions that form on dendrites, structural changes in dendritic spines of neurons are crucial in the implementation of synaptic plasticity, and for memory storage and neuronal connectivity^21^. Synaptic plasticity is influenced by the spatial pattern of synaptic inputs to the dendritic spines^47^. In vivo studies have shown that dendritic spines of the cortex in the VD mice were also impaired^48^. Consistent with the previously mentioned study, we also demonstrated that there was a significant reduction in the density of dendritic spines in the medial prefrontal cortex in the VD group when compared to the Con group. Enright et al.^49^ illustrated our result that hypoxic ischemic induced dendritic impaired in the brain is associated with cognitive impairment in VD rat model. Our study further demonstrated that the decrease in medial prefrontal cortex dendritic spines in the VD rats was ameliorated by treadmill exercise pretreatment. This phenomenon can be explained by the fact that exercise can exert neuroprotective effect by increasing cellular proliferation, dendritic complexity, and spine density in normal states^50^, and even to counter VD-induced neuron damage and dendritic injury^51^. In conclusion, treadmill exercise pretreatment can ameliorate VD-induced impairment of neuron and dendritic spines in the medial prefrontal cortex.

In our study, the results of transmission electron microscopy further demonstrated that there was a significant reduction in the density of synapses in the medial prefrontal cortex of VD rats, while treadmill exercise pretreatment increased the density of synapses in the medial prefrontal cortex of VD rats. In vivo imaging further demonstrated that VD rats showed remarkable damage of the synapse ultrastructure, such as the synaptic active zone, PSD thickness, and synaptic vesicles were significantly decreased^52,53^. The presynaptic processes of structural synaptic plasticity is reflected by the synaptic active zone, while PSD thickness reflects the postsynaptic neuronal mechanisms of structural synaptic plasticity^54,55^. In addition, synaptic vesicles mainly participated in information transmission and memory formation by releasing neurotransmitters^56^. Yang et al.^57^ demonstrated a significant reduction in the number of synaptic vesicles in VD rats. We further found that after VD, the dopamine and 5-HT levels in the medial prefrontal cortex are significantly decreased. However, these alterations were reversed by treadmill exercise pretreatment. Several body functions including reward, learning and executive functions are mediated by dopamine. Similarly, 5-HT is regarded as a modulator in various central nervous system functions, such as emotion, behavior, and cognition^58,59^. It has been practically proven that moderate exercise can increase neurotransmitters in both human^60^ and animal studies^37,61^, which are closely associated with learning memory and cognition. The studies performed by Li et al.^62^ indicated that voluntary wheel running induced switching from acetylcholine to gamma-aminobutyric acid expression in neurons in the caudal pedunculopontine nucleus. The beneficial effect of voluntary wheel running on motor skill learning could be blocked if viral vectors are used to override transmitter switching. Others studies have shown that cholinergic deficits and altered glutamatergic signaling that occur in VD patients may worsen cognitive impairment^63,64^. Our results supported this hypothesis as treadmill exercise pretreatment was found to alleviate VD-induced reduction in the number of synapses, and improve dopamine and 5-HT levels in the medial prefrontal cortex.

In summary, impaired structural synaptic plasticity in the rat medial prefrontal cortex is accompanied by recognition memory impairment and anxiety-like behavior under VD conditions. Such deficits can be ameliorated by treadmill exercise pretreatment. But this study still has some limitations, such as the behavioral results should be replicated using larger samples.

## Conclusion

Treadmill exercise pretreatment can ameliorate structural synaptic plasticity impairments of medial prefrontal cortex in VD rat and improved recognition memory.

## Data Availability

The datasets are available from the corresponding author on reasonable request.
